# Editorial: Rehabilitation care for clinically complex older adults across the continuum of care

**DOI:** 10.3389/fresc.2023.1328279

**Published:** 2023-11-20

**Authors:** Amanda Mofina, Caitlin McArthur

**Affiliations:** ^1^ School of Public Health Sciences, University of Waterloo, Waterloo, ON, Canada; ^2^ School of Physiotherapy, Dalhousie University, Halifax, NS, Canada

**Keywords:** rehabilitation, health systems, care continuum, complex, older adults

**Editorial on the Research Topic** Rehabilitation care for clinically complex older adults across the continuum of care

The number of people aged 60 years and older is increasing rapidly. The World Health Organization reported that this population will increase from 1 billion in 2020 to 1.4 billion in 2022 (1). Increased age is a risk factor for chronic illnesses and experiencing co-occurring chronic health conditions (2, 3). As we see the population of older adults increasing, and an increase in chronic health conditions, we will observe an unprecedented complexity in health care needs that span across the broader health determinants (4). As a result, it is expected that rehabilitation needs will increase across the health care continuum and the population that they serve will become increasingly complex (5).

The role of rehabilitation professionals (e.g., physiotherapists, occupational therapists and speech and language pathology therapists) includes optimizing peoples' health by addressing areas of physical, cognitive, environmental, and psychosocial dysfunction (5). Rehabilitation professionals play an important role across all health care sectors in assisting people to engage in meaningful activities within the various environments that they occupy. Existing literature has shown a disparity between rehabilitation needs and rehabilitation provision (5, 6). There is a growing need for rehabilitation services, and there are high rates of unmet rehabilitation needs related to unequal geographical distribution of services, affordability, and access-related reasons. Unmet rehabilitation needs are a global concern, with higher rates of unmet needs in middle- and low-income countries (6). Existing evidence has been based primarily on research using surveys with non-standardized measures for rehabilitation and rehabilitation needs, and therefore, findings may be an underestimation of the magnitude of current needs and makes international comparisons difficult (6).

It is important to establish a strong foundation of evidence to support the maintenance and implementation of rehabilitation services across various health care sectors using key health system evaluation indicators. Currently, there is limited rehabilitation research with a focus on health systems across various health care sectors. This research topic “Rehabilitation Care for Clinically Complex Older Adults Across the Continuum of Care” therefore aimed to highlight innovative research related to health policy, health systems, interventions, and rehabilitation evaluation models across the health care continuum for older adults.

There were four papers included in this Research Topic. Two articles focused solely on physiotherapy, one article focused on occupational therapy services, and one article considered physiotherapy, occupational therapy, and speech and language pathology therapists (Yamakawa et al., Turcotte et al., Alenezi et al., Shayo et al.). The studies examining the relationship between receipt of therapeutic intervention and subsequent functional outcomes revealed that receipt of therapy improved functional outcomes and some health-system level outcomes. Yamakawa et al. found that receipt of active occupational therapy improved functional outcomes and shortened hospital length of stay for those with acute stroke. The work conducted by Turcotte et al. examined how the interRAI community rehabilitation assessment (CRA) could be used with those receiving rehabilitation services in ambulatory care settings to monitor changes in key functional outcomes as well as for care planning, service and system evaluation, and benchmarking. Like the work conducted by Yamakawa et al. improvements post-rehabilitation therapy across key functional indicators were observed. The work conducted by Alenezi et al. examined the experience of physiotherapists use of compensatory strategies when working with individuals diagnosed with Parkinson's disease. Interestingly, this research highlights an evidence-to-practice gap by emphasizing the existing competencies and needs for physiotherapists working with people with a progressive chronic illness. Lastly, Shayo et al. described an innovative care delivery medium in a lower-middle income country to address the broader issue of accessibility. The authors integrated principles from two different care delivery adaptation models to demonstrate how the implementation of mobile phone rehabilitation services can be one solution to address the current issue related to accessing rehabilitation services. These articles add to the current evidence by elaborating on areas of expanding scope in response to an evolving health care system with high rehabilitative demands (Yamakawa et al., Turcotte et al., Alenezi et al., Shayo et al.).

The articles included in this Research Topic provide some key insights into innovative solutions and future research directions for older adults accessing rehabilitation services across the care continuum. There is, however, much work to be done with respect to further understanding the broader effect of rehabilitation across the health care continuum. We did not receive as many articles as we had hoped, and this may be attributed to the limited research that exists focusing on the system-level outcomes or rehabilitation care targeting those experiencing multiple chronic health conditions or health complexities (7).

There are promising developments in the rehabilitation field with respect to understanding and capturing system-level outcomes related to rehabilitation practice. Interestingly, one of the articles discusses an assessment that provides a significant source of data captured in a standardized way by trained healthcare professionals Turcotte et al. The interRAI CRA is one of many assessment instruments amongst a suite of established interRAI assessments. interRAI instruments are designed as an integrated system with common measures that rehabilitation professionals address (e.g., mobility, activities of daily living, instrumental activities of daily living, and falls) across different health care sectors (e.g., primary care, home care, long-term care, post-acute care) along the health care continuum (8). There are also measures across some of these assessments that capture current rehabilitation service provision as well as indicators of unmet rehabilitation needs (8, 9). Standardized measures of health system, service provision, and the effect of interventions are instrumental in situating rehabilitation services as an important component for aiding in health care transitions. These indicators also provide key health utilization information (e.g., amount of rehabilitation services received, receipt of other health care services such as nursing and social work, emergency department and hospitalization information, and long-term care use) beyond the typical biomedical markers of service utilization and effectiveness. With the aging population increasing, it is anticipated that the disparity between receipt of rehabilitation and unmet needs will continue to grow (1, 5, 6). The articles included in this Research Topic provide favourable findings related functional- and system-level processes and outcomes, and offer potential population-level data sources that situate rehabilitation professionals as crucial members of the health care team (Yamakawa et al., Turcotte et al., Alenezi et al., Shayo et al.). More evidence is needed, using comprehensive and standardized assessments that capture the scope and effect of the various practitioners involved in peoples' care journey.
